# Novel XIAP mutation with early-onset Crohn’s disease complicated with acute heart failure: a case report

**DOI:** 10.1186/s12872-023-03386-6

**Published:** 2023-07-21

**Authors:** Chendong Peng, Yuang Jiang, Xianhong Ou, Lei Liao, Chengying Yang, Qiao Zhou, Yan Wei, Lijia Chang, Xinrong Fan

**Affiliations:** 1 Department of Cardiology, The Affiliated Hospital of Southwest Medical University, 25 Taiping Street, Jiangyang District, Luzhou, 646000 Sichuan China; 2 Key Laboratory of Medical Electrophysiology of Ministry of Education and Medical Electrophysiological Key Laboratory of Sichuan Province, Collaborative Innovation Center for Prevention and Treatment of Cardiovascular Disease, Institute of Cardiovascular Research, Southwest Medical University, Luzhou, 646000 Sichuan China; 3 Prenatal Diagnosis Center, Shijiazhuang Obstetrics and Gynecology Hospital, Key Laboratory of Maternal and Fetal Medicine of Hebei Province, 16 Tangu-North Street, Shijiazhuang, 050000 Hebei China

**Keywords:** XIAP, Heart failure, Crohn’s disease, Thiamine deficiency

## Abstract

**Background:**

The X-linked inhibitor of apoptosis (XIAP) protein is encoded by the XIAP gene and is critical for multiple cell responses and plays a role in preventing cell death. XIAP mutations are associated with several diseases, primarily including hemophagocytic lymphohistiocytosis and inflammatory bowel disease (IBD). We report the clinical features and results associated with hemizygous mutation of the XIAP gene in a young male with Crohn’s disease complicated with acute heart failure.This 16-year-old patient ultimately died of heart failure.

**Case presentation:**

A young male of 16 years of age was initially diagnosed with Crohn’s disease based on evidences from endoscopic and histological findings. Although supportive care, anti-infective drugs and biologics were administered consecutively for 11 months, his clinical manifestations and laboratory indices (patient’s condition) did not improved. Additionally, the patient exhibited a poor nutritional status and sustained weight loss. Subsequently, acute heart failure led to the exacerbation of the patient’s condition. He was diagnosed with wet beriberi according to thiamine deficiency, but the standard medical therapy for heart failure and thiamine supplementation did not reverse the adverse outcomes. Comprehensive genetic analysis of peripheral blood-derived DNA revealed a novel hemizygous mutation of the XIAP gene (c.1259_1262 delACAG), which was inherited from his mother.

**Conclusion:**

A novel XIAP mutation (c.1259_1262 delACAG) was identified in this study. It may be one of the potential pathogenic factors in Crohn’s disease and plays an important role in the progression of heart failure. Additionally, thiamine deficiency triggers a vicious cycle.

## Background

The X-linked inhibitor of apoptosis (XIAP) protein is encoded by the XIAP gene and is critical for multiple cell responses. XIAP plays a role in preventing cell death by directly inhibiting caspase activities and regulating nucleotide binding oligomerization domain containing 2 (NOD2) in response to bacterial pathogens and tumor necrosis (TNF)-mediated survival, inflammation, and death signaling pathways [1, 2]. XIAP deficiency, caused by XIAP gene mutations, leads to a rare primary immunodeficiency disease with clinical phenotypes including hemophagocytic lymphohistiocytosis (HLH), lymphoma, splenomegaly, hypogammaglobulinemia and inflammatory bowel disease (IBD) [2]. Hematopoietic stem cell transplantation (HSCT) is considered the only curative treatment for numerous diseases, and it has been administered to patients with XIAP deficiency complicated with HLH and IBD. However, the efficacy and long-term prognosis are still unsatisfactory [3]. Furthermore, the diversity of disease phenotypes, breadth of severity and unpredictable onset between early infancy and adulthood make accurate and timely diagnosis and comprehensive and appropriate treatment more difficult in these patients [4]. Here, we report the first case of a young male with a novel XIAP mutation who suffered from early-onset Crohn’s enterocolitis and subsequently developed serious heart failure.

## Case presentation

**Fig. 1:**
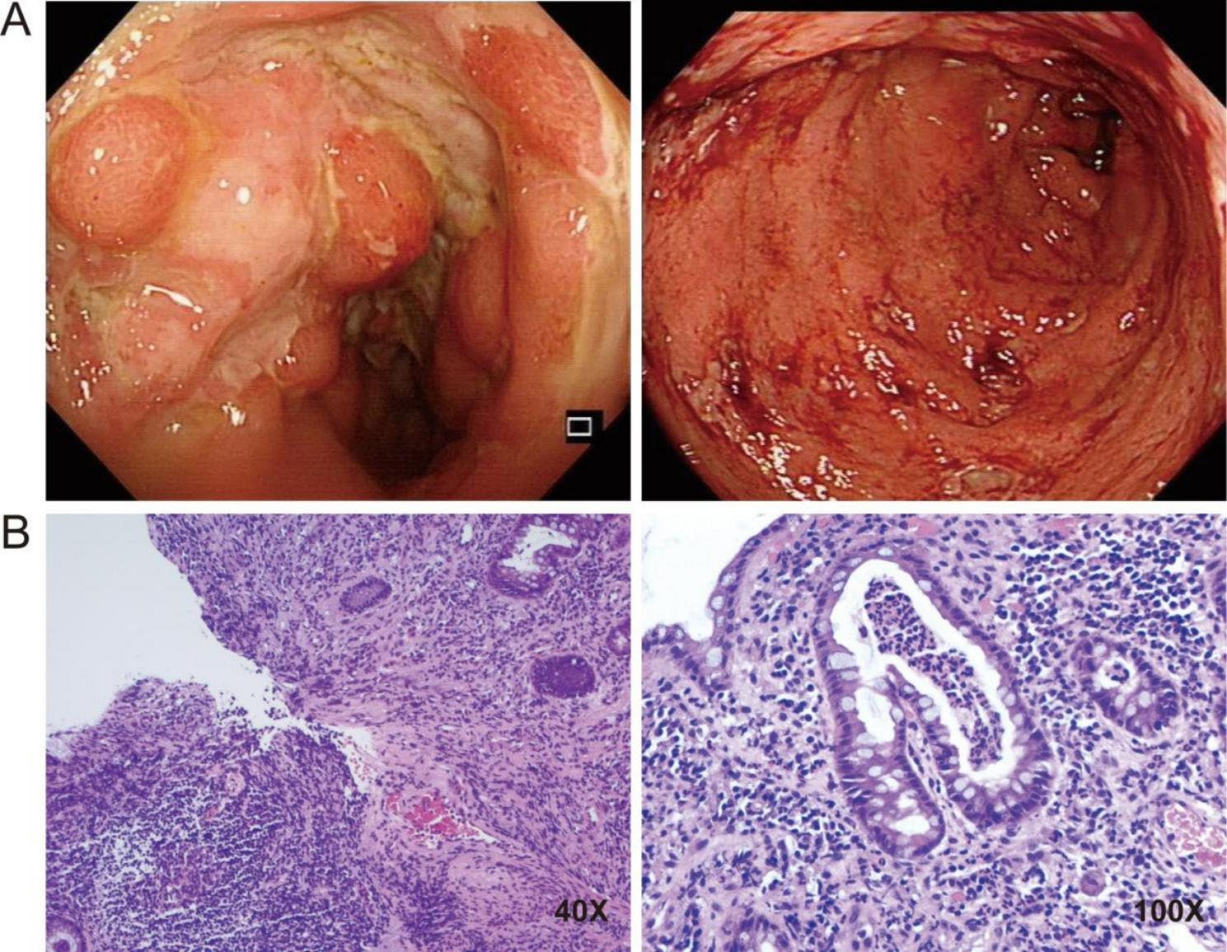
Endoscopy images and histologic analysis. **(A)** A colonoscopy revealed inflammatory colitis with erosions, aphthae and longitudinal ulcers. **(B)** Ileocolic biopsy indicated a large amount of lymphocytic, neutrophilic granulocytic and plasmacytic infiltration (low-power view), crypt abscesses (high-power view) and epithelial granulomas

A young male aged 16 years (body weight, 46 kg; height, 164 cm; BMI 17.1; ethnicity, Chinese) was admitted to the Department of Gastroenterology at our hospital for recurrent abdominal pain, diarrhea, and intermittent fever lasting 4 months. This patient had a surgical history of anal fistula. His family had no significant history of infectious disease, cancer, cardiovascular disease or autoimmune dysfunction. His conjunctiva and nail bed appeared pale, and the spleen was palpable at 3 cm below the costal margin. Detailed blood tests were performed during the whole medical period, as shown in Table 1. Anemia, hypoferritinemia, hypoalbuminemia, and positive serological results (IgG for Epstein‒Barr virus) were found. An abdominal CT scan showed significant thickening and edema of the ileocecocolic wall, luminal stenosis at the distal colon and celiac lymphadenectasis. A colonoscopy revealed inflammatory colitis with erosions, aphthae and longitudinal ulcers. In addition, ileocolic biopsy indicated a large amount of lymphocytic, neutrophilic granulocytic and plasmacytic infiltration, crypt abscesses and epithelial granulomas. The patient was diagnosed with Crohn’s disease on the basis of endoscopic and histological findings (Fig. 1). Anemia and fever were relieved by nutritional supplementation therapy and anti-infective drugs. Shortly thereafter, infliximab was administered as an alternative treatment for Crohn’s disease because of intolerance to corticosteroids and aminosalicylic acid. The cardiac assessment was conducted prior to the administration of biologics. Myocardiac biomarkers were normal and chest X-ray showed his heart shadow size was normal range at baseline. The patient had no limitations in general activities and did not exhibit symptoms such as palpitations, dyspnea, or angina. These findings were consistent with the guidelines for treating inflammatory bowel disease with anti-TNF agents. However, the patient’s clinical condition did not markedly improve after 7 cycles of biologics (4 doses of infliximab replaced by adalimumab) over 11 months. The patient had intermittent episodes of severe diarrhea, of which the occurrence ranged from 5 to 20–30 times per day. Subsequently, anemia, difficulty eating and diarrhea worsened when the patient decided to discontinue biologics due to the high cost. Although he was fed continuous enteral nutrition through a gastrojejunal tube, dramatic weight loss was observed over 17 months (weight, from 46 to 33 kg; BMI, from 17.1 to 11.1).

**Table 1:** Laboratory data at the time of first admission, 3 months later, 12 months later and last admission

Variable	Result				Reference rage
	First admission	3 months later	12 months later	Last admission	
Peripheral blood					
WBC (×10^9^/L)	6.59	4.25	2.92	3.93	3.5–9.5
RBC (×10^12^/L)	3.91	3.57	3.19	2.87	4.3–5.8
HGB (g/L)	82	76	85	77	130–175
MCV (fl)	71.10	73.10	88.70	91.90	82–100
MCH (pg)	20.90	21.30	26.50	26.70	27–34
MCHC (g/L)	294	291	299	291	316–354
PLT (×10^9^/L)	458	328	227	237	125–350
ESR (mm/hour)	104	105	-	24	0–26
Blood biochemistry					
ALT (U/L)	5.5	11.6	4.4	40.3	9–50
AST (U/L)	11.1	16.2	6.4	96.3	15–40
TP (g/L)	67.1	61.6	48.0	62.8	65–85
ALB (g/L)	32.4	33.6	25.4	32.6	40–55
GLO (g/L)	34.7	28.0	5.2	30.2	20–40
TBIL (µmol/L)	5.1	3.3	5.2	12.1	0–23
GGT (U/L)	37.3	15.5	8.7	187.5	10–60
ALP (U/L)	85.2	43.4	68.7	214.5	45–125
BUN (mmol/L)	3.75	3.39	4.33	12.56	3.1-8.0
UA (µmol/L)	242.8	196.6	106.9	356.6	208–428
Creatinine (µmol/L)	45.9	45.8	31.9	107.9	57–97
CRP (mg/L)	53.26	56.09	-	26.44	0–5
Electrolytes and minerals					
Potassium (mmol/L)	3.65	3.42	3.15	4.91	3.5–5.3
Sodium (mmol/L)	144.0	136.3	136.4	122.4	137–147
Chloride (mmol/L)	104.2	101.7	98.4	92.4	99–110
Calcium (mmol/L)	2.04	1.94	1.84	1.98	2.11–2.52
Magnesium (mmol/L)	-	-	-	0.56	0.75–1.02
Iron (µmol/L)	-	-	-	3.0	10.6–36.7
Cuprum (µmol/L)	-	-	-	28.2	11–24
Zinc (µmol/L)	-	-	-	6.1	11.1–19.5
Lipid profile					
TC (mmol/L)	3.20	-	2.63	3.21	2.9–5.18
TG (mmol/L)	1.26	-	1.10	1.57	0.4–1.7
HDL-C (mmol/L)	0.85	-	1.35	0.95	1.04–2.08
LDL-C (mmol/L)	2.00	-	1.07	1.88	1-3.37
Coagulation test					
PT (s)	14.3	14.8	15.0	14.1	11.0-14.5
PT-INR	1.09	1.16	1.17	1.08	0.80–1.2
PT %	87.0	78.0	78.0	87.0	70–130
APTT (s)	57.5	56.5	48.3	41.1	26–40
Fib (g/L)	4.87	4.40	3.99	4.63	2–4
D-dimer (ug/ml)	1.97	-	-	0.76	0-0.5
Anemia marker					
FER (ng/ml)	16.72	9.53	5.33	72.41	25.00-280.00
Vitamin B12 (pg/ml)	758.90	1296.00	1254.00	983.00	197.00-771.00
FA (ng/ml)	1.45	18.51	> 20.00	17.00	4.20–19.80
Cardiac marker					
TNT (µg/L)	0.006	-	0.013	0.089	0-1.014
MYO (µg/L)	21.00	-	21.20	85.80	0–72
CK-MB (µg/L)	0.378	-	0.965	0.879	0-4.87
NT-proBNP (ng/L)	-	-	-	18290.00	0–88
Virus detection					
EBV (copies/ml)	5.82E + 02	4.40E + 01	-	8.46E + 02	Not detected
HCMV (copies/ml)	< 1.00E + 01	Not detected	-	Not detected	Not detected
Vitamin					
Vitamin D (ng/ml)	6.00	11.20	-	7.50	> 30.00
Vitamin B1 (ng/ml)	-	-	-	2.108	2.41–9.03
Vitamin B2 (ng/ml)	-	-	-	14.262	1.00–19.00
Vitamin B3 (ng/ml)	-	-	-	74.428	10.00-158.00
Vitamin B5 (ng/ml)	-	-	-	115.288	37.00-147.00
Vitamin B6 (ng/ml)	-	-	-	3.759	3.00–30.00
Vitamin B7 (ng/ml)	-	-	-	1.903	0.22-3.00

Abbreviation: WBC: white blood cells; RBC: Red blood cells; HGB: Hemoglobin; MCV: mean corpuscular volume; MCH: mean corpuscular hemoglobin; MCHC: mean corpuscular hemoglobin concentration; PLT: platelets; ESR: erythrocyte sedimentation rate; ALT: alanine aminotransferase; AST: aspartate aminotransferase; TP: Total protein; ALB: Albumin; GLO: Globulin; TBIL: Total bilirubin; GGT: γ-glutamyl transpeptidase; ALP: alkaline phosphatase; BUN: blood urea nitrogen; UA: Uric acid; CRP: C-reactive protein; TC: Total cholesterol; TG: Triglyceride; HDL-C: High-density lipoprotein- cholesterol; LDL-C: low-density lipoprotein-cholesterol; PT: prothrombin time; PT-INR: prothrombin time international normalized ratio; PT %, prothrombin activity; APTT: activated partial thromboplastin time; Fib: fibrinogen; FER: Ferritin; FA: folic acid; TNT: troponin T; MYO: myohemoglobin; CK-MB: creatine phosphokinase-MB; NT-proBNP: N-terminal pro brain natriuretic peptide; EBV: Epstein-Barr Virus; HCMV: Human Cytomegalo Virus

**Fig. 2:**
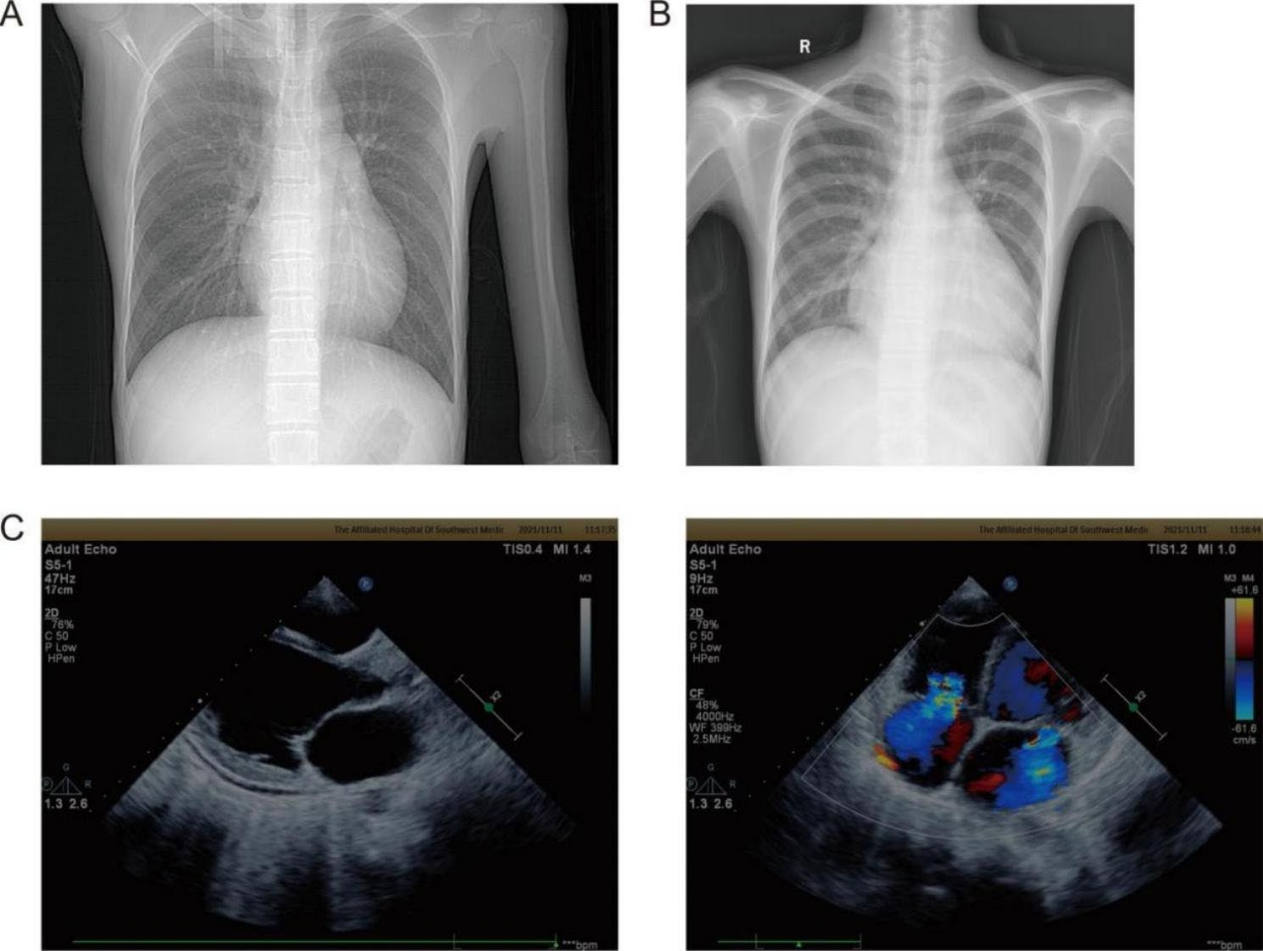
Chest X-ray and transthoracic echocardiography of the patient. **(A)** The chest X-ray of the patient showed a normal heart size at first admission. **(B)** The chest X-ray demonstrated an enlarged cardiac silhouette after 17 months. **(C)** Echocardiography revealed biventricular enlargement and moderate regurgitation of mitral and tricuspid valves after 17 months

Regretfully, this patient was readmitted to the Department of Gastroenterology for severe diarrhea accompanied by aggravating edema of the bilateral lower limbs and exertional and resting dyspnea. Moreover, he had numbness and weakness in his limbs and intermittent low-grade fever for 3 months. Auxiliary examinations were performed, and a marked increase in NT pro-BNP (18,604 ng/ml), slight elevation of troponin T (0.058 µg/L) and small multiple cavity effusion were observed. Furthermore, cardiac dilatation was found by chest X-ray (Fig. 2), and the same conclusion was verified by echocardiography, which revealed biventricular enlargement (LVd, 57 mm; RVd, 25 mm), prominent systolic dysfunction (EF, 31%) and moderate regurgitation of the mitral and tricuspid valves. Therefore, this patient was diagnosed with acute heart failure and transferred to our department.

In our department, the patient was slightly tachycardic (108 beats per minute) and hypotensive (90/48 mmHg). The other specialized examinations were performed. Twelve-lead electrocardiograms were compared between the initial and subsequent recordings, which showed augmentation of voltage in the QRS waves. The hemodynamic condition was acquired noninvasively by USCOM 1 A (Uscom Ltd, Sydney). The stroke volume index was 61 ml/m^2^, cardiac output was 7.1 L/min, cardiac index was 5.3 L/min/m^2^ and peripheral vascular resistance was 960 dyn ⋅ s^− 1^ ⋅ cm^− 5^, which indicated an abnormal physiological status of high output and low resistance. The patient and his family agreed to participate in a comprehensive genetic investigation. However, cardiac magnetic resonance imaging was not performed due to the patient’s critical condition. Large doses of loop diuretics, recombinant human brain natriuretic peptide, norepinephrine, and levosimendan were administered in turn. Not surprisingly, it was confirmed that the plasma vitamin B1 concentration was 2.108 ng/ml below the normal range, but no other soluble vitamin was affected by long-term malnutrition. Hence, this patient was soon injected intramuscularly with thiamine hydrochloride (300 mg per day). Unfortunately, the patient died after 4 days due to respiratory and circulatory failure caused by persistent high-grade fever and convulsions.

**Fig. 3:**
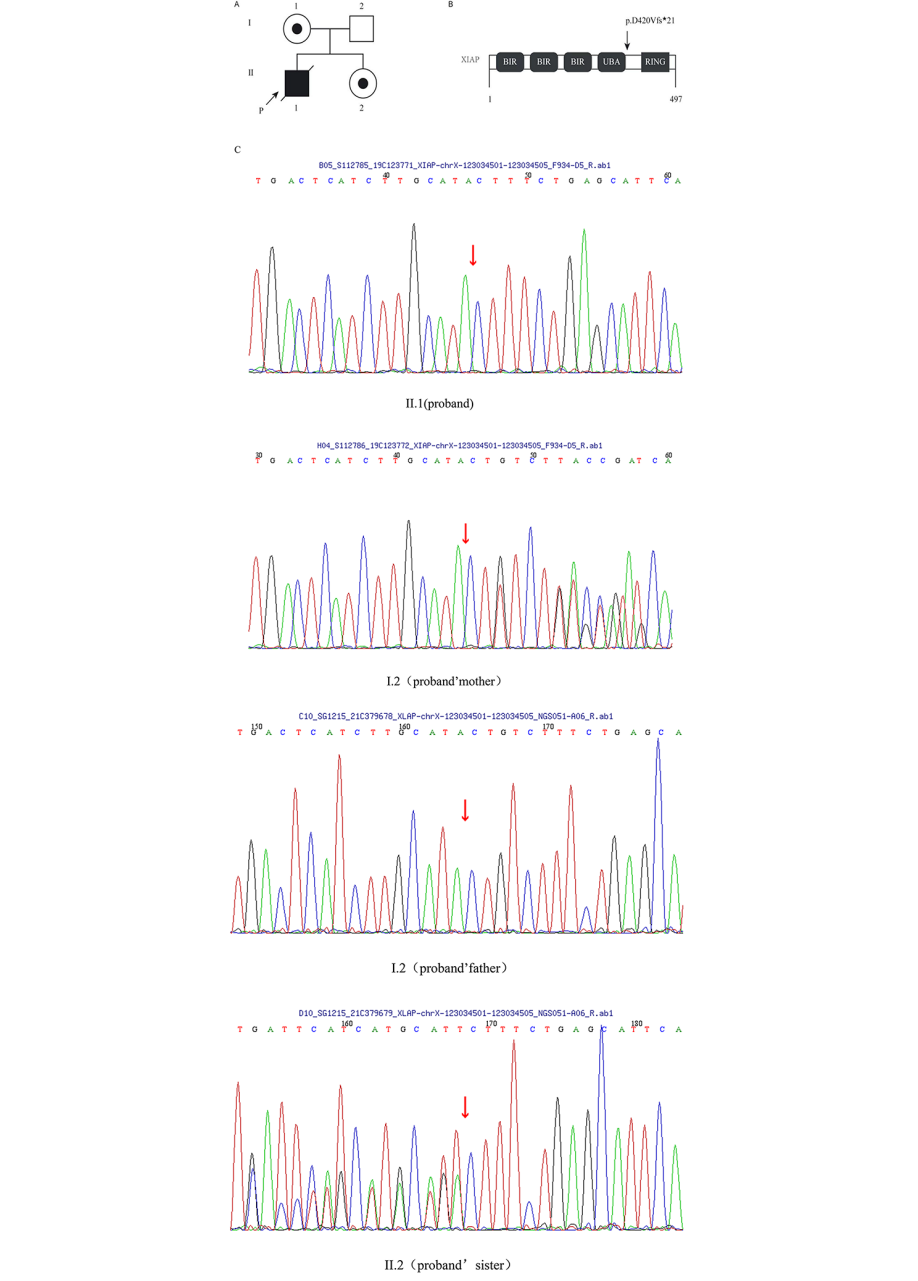
Mutation detection. **(A)** Pedigree of the family. The black box represents the affected individual. The diagonal bar indicates a deceased individual. Circles with black points identify female carriers. The arrow indicates the proband. **(B)** Sequence chromatograms of genomic DNA sequences of exon 6 of XIAP. **(C)** Protein domain structure of XIAP and localization of the mutation

Ten days after his death, his next-generation sequencing (NGS) results revealed a new mutation: c.1259_1262 delACAG (p.D420Vfs*21) in exon 6 of the XIAP gene. This mutation was predicted to be probably damaging by REVEL. According to the above evidence and the standards and guidelines of ACMG, this novel mutation was considered likely pathogenic. In addition, Sanger sequencing confirmed the presence of this mutation in the patient, the patient’s mother and younger sister, while it was not detected in the patient’s father. The family pedigree analysis is shown in Fig. 3.The clinical phenotypes of the patient’s relatives were normal.

## Discussion and conclusion

XIAP deficiency is rare and is estimated to affect approximately one in one million males. However, it is a severe and life-threatening inherited immune deficiency that was first described in 2006 [5–7]. It is characterized by extreme vulnerability to Epstein‒Barr virus (EBV) infection (66%). Common phenotypes of XIAP deficiency include HLH (60%) and inflammatory bowel disease (IBD) (25–30%). Additionally, there is a wide spectrum of other clinical characteristics, such as fever (8%), splenomegaly (14%), hypogammaglobulinemia (13%), liver disease (13%) and autoimmune disorders (9%). Most patients present with disease symptoms at very early ages, including infancy, childhood, adolescence and young adulthood [8]. In addition, Takeuchi and Chen et al. reported that XIAP mutations could lead to Takayasu arteritis and coronary artery dilation, respectively [9, 10]. However, no cases of a patient with an XIAP mutation complicated with cardiac disease have been reported thus far. In this case report, we describe for the first time a young male with early-onset Crohn’s disease and a heart failure course who was unresponsive to medical treatment options.

### Crohn’s disease related to XIAP deficiency

Since the first XIAP mutation case complicated with IBD was reported in 2011, [11] more than 30 cases have been reported in many other countries [12]. In our case, several characteristics, such as early age of onset (16 years old), surgical history of anal fistula, typical endoscopic and histological findings, noneffective treatments by glucocorticoids and biologics (infliximab or adalimumab), and poor prognosis, which indicated that intractable Crohn’s enterocolitis was potentially caused by genetic variations. The post-hoc analysis of whole-exon sequencing confirmed that the novel frameshift mutation in exon 6 of the XIAP gene was attributed to distinctive phenotypes. XIAP is critical for downstream signaling in Crohn’s disease associated with susceptibility proteins and essential for signal transduction of NOD1 and NOD2. NOD1 and NOD2 are intracellular pattern recognition receptors involved in the innate immune host defenses [13]. Various mutations in the XIAP gene, such as frameshift mutations, missense mutations, deletions of exons/amino acids, nonsense mutations and splice-site mutations, are distributed throughout the entire gene. These mutations may lead to cDNA depletion or a low copy number, which may be associated with the instability of the mutant version of mRNA. In addition, these mutations can also modify the structure and function of the XIAP protein when a single amino acid or a sequence is replaced or deleted. In our case,the genetic analysis of XIAP revealed a frameshift mutation and replacement of partial cDNA, which might lead to significant XIAP deficiency even though it was not verified by western blotting. A previous study indicated that various XIAP functions are associated with three baculovirus IAP repeats (BIRs), a ubiquitin-associated (UBA) domain and truly interesting new gene (RING) finger domains, [14] which are required for the ubiquitination of proteins to promote cell survival. The abnormal structure in the RING domain caused by a mutation in exon 6 disturbs intestinal homeostasis by interacting with the abnormally activated T cells and invariant natural killer (iNK) cells. This was consistent with Yang’s report of a patient with HLH and IBD with a frameshift mutation in exon 6 of the XIAP gene [1]. Although some studies have indicated that XIAP deficiency is an important cause of IBD, the gene penetrance was not very high in the observed population (less than 30%). A Chinese study reported that only 1 of 22 patients with XIAP mutations developed IBD, while no other patients or carrier mothers suffered from colitis or other intestinal manifestations [8]. Some researchers considered that XIAP-BIR2 mutation interfered with the XIAP and receptor interacting serine/threonine kinase 2 (RIPK2) interaction, resulting in the aberrant activation of NF-kappa B and promotion of apoptosis in response to various stimuli [13]. This may explain why patients with XIAP deficiency had manifestations of colitis. Nevertheless, other investigators indicated that IBD with XIAP malfunction were not associated with mutations in particular domains [15]. In addition, recent studies have indicated that environmental factors, such as infection with EBV or cytomegalovirus (CMV), dietary mode and microbial dysbiosis in the gut, might contribute to the differences in clinical phenotypes in patients with XIAP mutations [16]. In our case, this young male developed intractable Crohn’s disease rapidly over 2 years, which might be due to persistent EBV infection and a high-calorie, low-fiber diet in childhood.

### Thiamine deficiency, beriberi and acute heart failure

Thiamine, also called vitamin B1, is a water-soluble vitamin that is essential for the functioning of multicellular organisms ,which is a cofactor for multiple steps in glycolysis and the oxidative decarboxylation of carbohydrates. It also plays a role in the immune system [17]. Although thiamine deficiency is not common in modern society, it is most epidemic in patients with chronic alcoholism or those with poor nutritional status, including poor oral intake or extreme dieting, prolonged enteral nutrition, severe chronic diarrhea, or following bariatric surgery [18]. Thiamine deficiency can result in complications such as cellular damage and endothelial dysfunction and increase oxidative stress. Severe thiamine deficiency, lasting more than 3 months, can lead to beriberi, which has both dry and wet manifestations. Dry beriberi is characterized by peripheral neuropathy, sensory and motor deficiencies of the extremities and muscle pain with atrophy. Wet beriberi is predominantly associated with cardiovascular symptoms and heart failure [19].

In our case, this young patient suffered from inadequate nutrition caused by severe chronic diarrhea and prolonged enteral nutrition, which could result in the excessive loss and incomplete absorption of vitamins and minerals. As expected, thiamine deficiency was revealed by mass spectrometry of the serum from this patient. In addition, he complained of numbness in his limbs, which might be associated with peripheral neuropathy. However, this was not confirmed by electromyogram or muscle biopsy. Notably, the cardiac complications were more serious than the neural complications in this patient. Cardiomegaly and heart failure were rapid and intractable. The hemodynamic parameters demonstrated high-output heart failure and low vascular resistance, which was consistent with the pathophysiological basis of wet beriberi [19]. Based on the above evidence, we considered that his diagnosis of Crohn’s disease complicated with wet beriberi had been confirmed. However, thiamine supplementation was not an effective therapy for this patient, which was a conflicting result compared with Jazayeri’s report [20]. It was possible that short-term thiamine supplementation was not enough to recover organ function,besides, the use of a large dose of loop diuretics might lead to more the loss of thiamine in the therapeutic process. In Dinicolantonio’s study, the prevalence of thiamine deficiency varied from 21 to 98% in patients with heart failure, thiamine deficiency was also more prevalent in patients with diuretic use [21]. Furthermore, hereditary variation might be a potential factor that could compromise the curative effect.

### Relationship between XIAP and heart failure

Heart failure is a clinical syndrome with a high mortality and increased prevalence in populations with cardiovascular risk factors, which are classified as acquired pathology and hereditary predisposition [22]. The rapid and continual progress of molecular biological techniques over the past two decades, along with the increased availability of various genetic testing methods, has led to a significant improvement in our understanding of the genetic architecture of heart failure. To date, many candidate genes have been attributed to important factors,which are related to the occurrence and development of heart failure, including encoding sarcomeric and cytoskeleton proteins (e.g., TTN, MYH7, and MYBPC3), calcium-handled proteins (e.g., PLN and RYR2), metabolic enzymes (e.g., GLA and PRKAG2) and transmembrane proteins (e.g., LAMP2 and SCN5A) [23], while there are few reports on the relationship between genes involved in immune regulation and cardiomyopathy. We speculated that XIAP deficiency might be a potential pathogenic factor that led to severe heart failure in our case. Two small-sample clinical studies showed that apoptotic inhibitors, including XIAP and other antiapoptotic factors, were significantly down-regulated in failed donor hearts compared with non-failed donor hearts [24, 25]. These results indicated that XIAP was closely related to myocardial apoptosis during the development of heart failure. In addition, Sun et al. and Cheng et al. reported that myocardial injury was attenuated by regulation of lncRNA and microRNA in sepsis and ischemia/reperfusion injury models, respectively These short-chain RNAs regulated the expression of XIAP to prevent cardiomyocyte death [26, 27]. Furthermore, empagliflozin, a novel drug for heart failure, prevented cardiomyocyte cell death by restoring the expression of XIAP [28]. Although there has been no report on hereditary cardiomyopathy induced by XIAP mutations to date, we could draw a reasonable conclusion that XIAP deficiency might play an important role in the process of heart failure and thiamine deficiency triggered a vicious spiral.In our case, administration of anti-TNF drugs was confounding factor for diagnosis of heart faiulure. Currently, there were controversial topics regarding the impact of anti-TNF agents on cardiac function. Basic studies indicated that the anti-TNF agents (infliximab) could improve left ventricular function and alleviate phenotype of heart failure in transgenic mice with TNF-αoverexpression [29, 30]. Clinically, incidence of heart failure induced by anti-TNF agents was extremely low. Two rare case reports described two patients with Crohn’s disease who developed heart failure after use of infliximab and adalimumab, respectively. However, the two patients’ heart failure symptoms and cardiac function significantly improved after discontinued anti-TNF agents and received treatment with anti-heart failure medications [31, 32]. Their reports were different from our results that heart failure in this young male was still progressing when the anti-TNF agents were discontinued for 6 months. Considering the patient’s genetic testing results and evidence of thiamine deficiency, the likelihood of anti-TNF agents causing heart failure in this patient was minimal. Of course, more detailed evidences are needed.

### Lessons learned and conclusion

Overall, this was not a successful case, as the young patient failed to respond to conventional treatment and rapidly died from severe complications. In conclusion, we learned several lessons. First, precise molecular genetic testing should be performed as early as possible, because a delayed or false diagnosis can lead to a poor prognosis in those with a hereditary disease. Second, it is recommended that hematopoietic stem cell transplantation should be performed as soon as possible for patients with HLH or aplastic anemia, because the outcome without HSCT is extremely poor [33]. However, a high incidence rate of acute graft-versus-host disease was observed. Shintaro et al. reported that pathological symptoms and dysbiosis of the gut microbiota were markedly improved after HSCT in 19 patients suffering from refractory IBD, but 4 patients died from complications [16]. Early HSCT might be a promising and effective therapy for these patients. Third, several studies have shown that micronutrient deficiencies in long-term Crohn’s disease patients are not common and usually result in a combination of reduced dietary intake, disease-related malabsorption and catabolic state [20, 34]. Opportune supplementation with vitamins and minerals might prevent multiorgan function disturbances caused by nutrient deficiencies. Finally, the comprehensive pathogenesis of early-onset Crohn’s disease related to XIAP mutations and differences between genotypes and phenotypes will need to be elucidated by more intricate experiments in the future.

## Data Availability

All data supporting the conclusions are presented in the manuscript.
